# Low and High Molecular Weight FGF-2 Have Differential Effects on Astrocyte Proliferation, but Are Both Protective Against Aβ-Induced Cytotoxicity

**DOI:** 10.3389/fnmol.2019.00328

**Published:** 2020-01-24

**Authors:** Xi Chen, Zhaojin Li, Yong Cheng, Elissavet Kardami, Y. Peng Loh

**Affiliations:** ^1^Key Laboratory of Ethnomedicine for Ministry of Education, Center on Translational Neuroscience, College of Life and Environmental Sciences, Minzu University of China, Beijing, China; ^2^Section on Cellular Neurobiology, Eunice Kennedy Shriver National Institute of Child Health and Human Development, National Institutes of Health, Bethesda, MD, United States; ^3^Institute of Cardiovascular Sciences, St. Boniface Hospital Albrechtsen Research Centre, University of Manitoba, Winnipeg, MB, Canada

**Keywords:** basic fiboblast growth factor, astrocyte, proliferation, amyloid beta, Cyclin D1

## Abstract

Astrocytes are the most abundant type of glial cells in the brain, and they play a key role in Alzheimer’s disease (AD). Fibroblast Growth Factor-2 (FGF-2) has been implicated as a potential therapeutic agent for treating AD. In the present study, we investigated the protective effects of low molecular weight (LMW; 17 KDa) and high molecular weight (HMW; 23 KDa) forms of FGF-2 on Aβ_1–42_-induced toxicity, and proliferation in astrocytes. We show that both isoforms of FGF-2 have similar protective effects against Aβ_1–42_-induced cytotoxicity in primary cultured cortical astrocytes as measured by Lactate Dehydrogenase (LDH) release assay. Additionally, 17 KDa FGF-2 significantly promoted astrocyte proliferation as measured by Trypan Blue, DRAQ5 and 5-ethynyl-2’-deoxyuridine (EdU) staining, but not 23 kDa FGF-2. Furthermore, our results demonstrated that AKT signaling pathway was required for the protective and proliferative effects of FGF-2. Downstream effector studies indicated that 17 kDa FGF-2 promoted astrocyte proliferation by enhanced expression of c-Myc, Cyclin D1, Cyclin E. Furthermore, our data suggested that Cyclin D1 was required for the proliferative effect of LMW FGF2 in astrocytes. Taken together, our findings provide important information for the similarities and differences between 23 kDa and17 kDa isoforms of FGF-2 on astrocyte survival and proliferation.

## Introduction

Alzheimer’s disease (AD) is the most prevalent type of dementia and is characterized by the progressive decline, and ultimately, loss of multiple cognitive functions (Ai et al., 2019; Das et al., 2019). The characteristic pathologic hallmarks, including neuritic plaques, synaptic loss (Katsouri et al., 2015), neurofibrillary tangles, and deposits of Aβ have been observed in the brain of AD patients (Granadillo et al., 2017). Aβ is believed to be a pivotal mediator of neuronal degeneration, leading to impaired cognitive function (Mattson, 2004; Hardy, 2006). It is, therefore, imperative to search for drugs that target Aβ toxicity.

Aβ is a peptide of 39–43 amino acids and has been shown to have a wide range of toxic effects *in vitro* and *in vivo*, including excitotoxicity, mitochondrial alterations, synaptic dysfunction, altered calcium homeostasis and oxidative stress (Carrillo-Mora et al., 2014). Most researchers have focused on the Aβ_1-42_ form which has been found to be more aggregation-prone (Finder et al., 2010; Hubin et al., 2014) and is considered to be more closely linked with AD pathogenesis than the Aβ_1-40_ form (Harasta and Ittner, 2017).

Astrocytes, the most abundant glial cell type in the brain, have various functions in maintaining brain physiology (Kimura et al., 2006), including support of endothelial cells that form the blood-brain barrier, provision of nutrients to neurons and maintenance of extracellular ion balance. Loss of astrocyte function contributes to the aging of the brain and pathogenesis of neurodegenerative diseases (Herholz et al., 2018), and reactive astrocytes are closely related with plaques and neurofibrillary tangles in AD (Serrano-Pozo et al., 2011).

**Figure 1:**
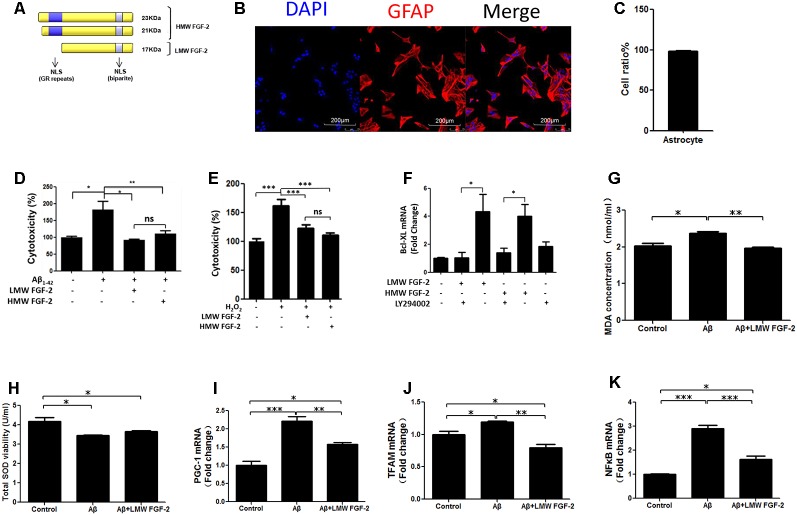
Fibroblast Growth Factor-2 (FGF-2) protects rat cortical astrocytes against Aβ_1–42_-induced cytotoxicity. **(A)** Schematic depicting the different isoforms FGF-2 in rat, resulting from alternative translation initiations; nuclear localization sequences (NLS); GR, Glutamic acid-Arginine. **(B)** Astrocytes were stained by DAPI (blue) and GFAP (red). **(C)** Quantification of astrocyte purity. **(D)** Lactate Dehydrogenase (LDH) activity in the primary cultured astrocytes treated with or without 20 μM Aβ_1–42_. Note that increased cytotoxicity after Aβ_1–42_ treatment is significantly attenuated by 10 ng/ml purified FGF-2. **(E)** LDH activity in primary cultured astrocytes treated with 200 μM H_2_O_2_ (oxidative stress) with or without 10 ng/ml FGF-2. Note that both isoforms of FGF-2 protected astrocytes against H_2_O_2_ damage, with no significant difference of protective effects between low molecular weight (LMW) and high molecular weight (HMW) FGF-2. **(F)** qRT-PCR analysis of Bcl-XL expression in primary cultured astrocytes treated with LY294002 and two isoforms of FGF-2. Note that the expression of Bcl-XL was down-regulated by LY294002 in the primary cultured astrocytes, indicating that Bcl-XL is a downstream effector of AKT. **(G)** The malondialdehyde (MDA) concentrations after various treatments in the astrocytes.** (H)** The superoxide dismutase (SOD) activities after various treatments in the astrocytes. **(I–K)** qRT-PCR analysis of PGC-1, TFAM and NFκB expression in primary cultured astrocytes treated with Aβ_1–42_ in the presence or absence of LMW FGF-2, *n* = 5. *p < 0.05, **p < 0.01, ***p < 0.001. ns, non-significance.

Fibroblast growth factors (FGFs) can supply trophic support, increase proliferation (Katsouri et al., 2015) and prevent apoptosis by acting against the activation of the mitochondrial apoptosis pathway (Sa-Nguanmoo et al., 2016). Fibroblast growth factor-2 (FGF-2) is a heparin-binding growth factor and has a homologous central core of 140 amino acids (Li et al., 2003). In the central nervous system, FGF-2 is expressed in various cell types including astrocytes, microglia, and neurons (Woodbury and Ikezu, 2014) and is considered to have angiogenic, mitotic, and neurotrophic effects (Kiyota et al., 2011; Steringer and Nickel, 2018). FGF-2 also has several isoforms resulting from alternative initiations of mRNA translation, with isoform-selective biological activities: a 17 KDa cytoplasmic isoform and two higher molecular weight isoforms: 21 and 23 KDa (Liu et al., 2015; Meo Burt et al., 2016; Wang et al., 2018; Figure 1A). The structure of 17 KDa FGF-2 has been determined by several groups, and the backbone of FGF-2 has a trigonal pyramidal structure with 12 antiparallel beta-sheets, and helix-like structures have been found at residues 131–136 (Liao et al., 2009; Liang et al., 2018). The 23 kDa form has two GR (Glu/Arg) repeats that work as Nuclear Localization Sequences (NLS), whereas the 17 kDa form has one. Different domains contained in the different isoforms of FGF-2 can control the subcellular localization and influence their function. For example, the 17 kDa FGF-2 is mainly found in the cytoplasm and the 23 kDa isoform is localized mainly to the nucleus (Rhoads et al., 2000; Müller-Ostermeyer et al., 2001). However, both 17 kDa and 23 kDa were found in the extracellular space of various types of cells (Santiago et al., 2011; Cheng et al., 2015), indicating that both isoforms can exert biological activities by interacting with plasma membrane receptors. Although the mechanism underlying the release of FGF-2 is still not fully understood, it is suggested that membrane vesicle shedding can mediate FGF-2 release from cells (Taverna et al., 2003), and signaling by FGF-2 occurs through the high-affinity tyrosine kinase receptors, FGFRs (Zhou et al., 2019).

Although various studies have shown neuroprotective properties of FGF-2 in neurons, there are limited studies on the role of FGF-2 in glial cells. Moreover, the function of extracellular-acting high molecular weight (HMW) 23 KDa FGF-2 has not been well established in the nervous system. In the current study, we investigated the effects of 17 KDa and 23 KDa FGF-2 in astrocyte proliferation and protection against Aβ toxicity, and the mechanisms underlying them. We found that while both isoforms of FGF-2 had similar protective effects against Aβ_1–42_ induced toxicity in cortical astrocytes, only the 17 KDa FGF-2 promoted astrocyte proliferation.

## Materials and Methods

### Animals

Pregnant rats were purchased from Taconic Farms, Derwood, MD, and Vital River Laboratory, Beijing, China. All animals were given food and water *ad libitum* in humidity and temperature-controlled room under a 12 h light:dark cycle. All the methods were carried out in accordance with the guidelines approved by the Animal Care and Use Committee NICHD, NIH, and the Animal Care and Use Committee of the Minzu University of China.

### Primary Astrocyte Culture

Brains from postnatal day 1 rats were removed. The cortex was dissected and digested by 2 ml trypsin (0.25%) for 15 min at 37°C, which was then inactivated by 3 ml of 10% Fetal Bovine Serum (FBS). The tissue was triturated by a pipette to make a homogenous mixture, which was passed through a cell strainer to remove undissociated tissue. The cells were centrifuged for 5 min at 1,800× *g*, and the supernatant was discarded. The cell pellet was resuspended in complete DMEM media containing antibiotics (1% Penicillin-Streptomycin) and 10% FBS. The cells were then plated in a flask.

### Treatment of Cortical Astrocytes With Aβ_1–42_ With or Without LMW (17 KDa) and HMW (23 KDa) FGF-2

Primary cultured cortical astrocytes were treated with 20 μM Aβ_1–42_ (purchased from PEPTIDE 2.0 Inc., Chantilly, VA, USA) in serum-free condition, with or without 10 ng/ml purified recombinant LMW and HMW FGF-2, obtained as previously described (Santiago et al., 2011). Cell cytotoxicity was tested by the Lactate Dehydrogenase (LDH) release assay after 24 h of treatment and cell viability was tested by water-soluble tetrazolium (WST-1). Cell viability or cell cytotoxicity were assayed by WST-1 or LDH assays (see below).

### Malondialdehyde (MDA) and Superoxide Dismutase (SOD) Assay

Primary cultured cortical astrocytes were treated with 20 μM Aβ_1–42_ in serum-free conditions, with or without 10 ng/ml purified recombinant LMW FGF-2. Cell supernatants were collected after 24 h of treatment, and malondialdehyde (MDA) concentration and total superoxide dismutase (SOD) activity were detected by MDA and SOD kits according to the manufacturer’s protocols (Nanjing Jiancheng, Nanjing, China).

### Treatment of Cortical Astrocytes With LMW and HMW FGF-2 With or Without FGFR1 Inhibitors

Primary cultured cortical astrocytes were first incubated with FGFR1 inhibitor PD166285 (0.1 μM, Sigma) or SU5402 (10 μM, Sigma) for 30 min after which the LMW or HMW FGF-2 (10 ng/ml) was added to the wells, and incubated for 24 h. The astrocytes were tested for cell viability and cytotoxicity by the WST-1 and LDH assay, respectively (see below).

### Treatment of Cortical Astrocytes With LMW and HMW FGF-2 With or Without ERK and AKT Inhibitors

Primary cultured cortical astrocytes were incubated with LMW and HMW FGF-2 (10 ng/ml) for 30 min in serum-free condition, then cells were harvested and lysed, and Western blot was used to detect p-ERK and p-AKT. In other experiments, primary cultured astrocytes were preincubated with or without the ERK inhibitor, U0126 (5 μM, Cell Signaling), or the AKT inhibitors LY294002 (10 μM, Cell Signaling) and MK-2206 (30 μM, Selleck) for 30 min after which 10 ng/ml FGF-2 was added, where indicated, and incubated for a further 30 min. The cells were harvested directly using loading buffer and the corresponding cell lysates were analyzed by Western blot for p-ERK, total-ERK and p-AKT, total-AKT. To determine whether the ERK and AKT pathways were involved in the FGF-2 dependent survival, U0126 and LY294002 were used to inhibit MEK/ERK and PI-3K/AKT signaling pathways, respectively. Primary cultured astrocytes were preincubated with ERK and AKT inhibitors for 30 min after which the low or HMW FGF-2 (10 ng/ml) was added to the culture wells, and incubated for 24 h. The astrocytes were tested for cell viability and cytotoxicity by the WST-1 and LDH assays, respectively (see below).

### WST Assay for Cell Viability

The viability of astrocytes was determined by the WST (soluble form of MTT) Cell Proliferation (Clontech) assay in a 96 well plate according to the manufacturer’s protocol.

### LDH Release Assay for Cell Cytotoxicity

The cytotoxicity of astrocytes after various treatments was evaluated by the extent of the release of LDH. This was achieved with a CytoTox 96 Non-radioactive Cytotoxicity Assay Kit according to the manufacturer’s instructions (Promega, Madison, WI, USA).

### Trypan Blue Staining

The astrocyte numbers were first determined directly by trypan blue staining. A 1:1 dilution of cell suspension was prepared using a 0.4% trypan blue solution (Invitrogen, Waltham, MA, USA) that stains non-viable cells. A cell counting chamber slide (Invitrogen, Waltham, MA, USA) was carefully filled with the cell suspension and the cell number was counted using an automated cell counter (Invitrogen, Waltham, MA, USA).

### DRAQ5 Assay

The astrocyte numbers were also determined by DRAQ5 assay. DRAQ5 is an anthraquinone dye with high affinity for double-stranded DNA that can label live or fixed/dead cells. This was achieved with Far-red Fluorescent Live-cell Permeant DNA dye according to the manufacturer’s instructions (Abcam, Cambridge, UK). The red fluorescent signals were quantified by the Odyssey infrared imaging system and software v2.1 (LI-COR Inc., Lincoln, NE, USA).

### Cyclin D1 Transfection

The Cyclin D1 siRNA and negative control (NC) were purchased from Thermo Fisher Scientific. Astrocytes were transfected with NC or Cyclin D1 siRNA using Lipofectamine 3000 (Thermo Fisher Scientific, Waltham, MA, USA) following the manufacturer’s protocol. The experiments were performed 48 h after transfection.

### RNA Extraction and qRT-PCR

Extraction of total RNA from treated astrocytes was performed using an RNeasy Mini Kit (QIAGEN) and complementary DNA (cDNA) was synthesized from total RNA in two stages as described by the standard protocols with SYBR Green Master Mix (Thermo Fisher Scientific, Waltham, MA, USA) and subsequently used to measure Bcl-XL, c-Myc, Cyclin D1, Cyclin E, β-Catenin, PGC-1, NFκB, and TFAM. The gene expression was normalized to the housekeeping gene 18S. The sequences of primers were listed below.

**Table:** 

Gene name	Sequence (5′-3′)
Bcl-xl-R	ACATCAAAACCAAGGCAAGC
Bcl-xl-F	GGGACCCTAATTACCCCTGA
c-Myc-R	TCATCTGCTTGAACGGACAG
c-Myc-F	CCAGATCCCTGAGTTGGAAA
Cyclin D1-R	GCAAGAATGTGCCAGACTCA
Cyclin D1-F	GGAGATGTGGGTCTCCTTGA
Cyclin E-R	TCTGCATCAACTCCAACGAG
Cyclin E-F	ATGTCCAAGTGGCCTACGTC
β-Catenin-R	GAGCTTGCTTTCCTGATTGC
β-Catenin-F	ACGCTGCATAATCTCCTGCT
18S-R	CTGATCGTCTTCGAACCTCC
18S-F	CTCTTAGCTGAGTGTCCCGC
NFκB-R	GGTCCCGTGAAATACACCTC
NFκB-F	CCGAGTAAACCGGAACTCTG
PGC-1-F	TGCAGCCAAGACTCTGTATG
PGC-1-R	TCAATCCACCCAGAAAGCTG
TFAM-F	GCAGTGGTGAATTGTTCTGC
TFAM-R	GTAAAGCCCGGAAGGTTCTT

### Western Blot Analyses

Soluble protein lysates of cortical astrocytes in culture were prepared by homogenizing the cells in T-protein extraction reagent (Thermo Fisher Scientific, Waltham, MA, USA) supplemented with 0.1% Triton X-100 (Sigma) and Protease Inhibitor Cocktail (Roche, Basel, Switzerland). The samples were then prepared with a reducing agent (Invitrogen, Waltham, MA, USA) and NuPAGE LDS Sample Buffer (Invitrogen, Waltham, MA, USA), and protein samples were analyzed by standard Western blot procedures using nitrocellulose. Protein bands were visualized and quantified by the Odyssey infrared imaging system and software v2.1 (LI-COR Inc., Lincoln, NE, USA) Monoclonal mouse anti-β-Catenin antibody (1:1,000) was from Santa Cruz. Monoclonal rabbit anti-Cyclin D1 antibody (1:200) was from Abcam. Polyclonal rabbit anti-c-Myc antibody (1:1,000) was from BBI Life Sciences. Polyclonal rabbit anti-AKT antibody (1:5,000), polyclonal rabbit anti-ERK antibody (1:5,000), monoclonal mouse anti-pERK antibody (1:5,000), monoclonal mouse anti-pAKT (Ser 473) antibody (1:5,000), monoclonal rabbit anti-pGSK-3β (Ser 9) antibody (1:3,000), monoclonal mouse anti-GSK-3β antibody (1:3,000) and monoclonal mouse anti-β-Actin antibody (1:5,000) were from Cell Signaling.

### Simon Western Blot Analysis (ProteinSimple, CA, USA)

In some cases, western blot analysis was performed using a capillary-based automated system^1^ by following the manufacturer’s protocol.

### Immunofluorescence

For immunofluorescence, cells plated on chamber slides were fixed with 4% Paraformaldehyde at room temperature for 15 min. Following washing three times with PBS, cells were blocked by PBS containing 0.3% Triton X-100 and 10% goat serum for 1 h. Then cells were incubated in primary antibody solution overnight at 4°C. The primary antibody used was rabbit anti-GFAP antibody (1:1,000, Sigma). After three times washing with PBS, cells were incubated with goat anti-rabbit secondary antibody (1:2,000) for 1 h at room temperature. After washing the secondary antibody, 0.5 ng/ml of DAPI (Sigma) was added in the chamber for 30 min to stain the cell nuclei. The cells were examined with a digital eclipse 80i microscope (Nikon, Tokyo, Japan). 5-ethynyl-2′-deoxyuridine (EdU) staining was achieved by the Click-iT Plus EdU Alexa Fluor 488 Imaging kit (Thermo Fisher Scientific, Waltham, MA, USA) and performed according to manufacturer’s instructions.

### Statistical Analysis

Data were analyzed by one-way analysis of variance (ANOVA) followed by a *post hoc* test for multiple group comparisons, and Student’s *t*-test was used for comparison of two groups. Statistical significance was set at ***p < 0.001; **p < 0.01; *p < 0.05.

## Results

### FGF-2 Protects Rat Cortical Astrocytes Against Aβ_1–42_-Induced Cytotoxicity and Oxidative Stress

To determine whether FGF-2 (Figure 1A) protects astrocytes against Aβ_1–42_ toxicity, 20 μM Aβ_1–42_ with or without 10 ng/ml LMW and HMW FGF-2 was added to the media of the cultured astrocytes and incubated for 24 h, and the purity of cultured astrocytes was above 95% (Figures 1B,C). As shown in Figure 1D, Aβ_1–42_ treatment significantly increased cytotoxicity, and FGF-2 supplementation significantly decreased cytotoxicity. There was no difference in the protective effect between the LMW and HMW FGF-2. This protective effect was further investigated by adding both isoforms of FGF-2 to astrocytes subjected to oxidative stress induced by 200 μM H_2_O_2_ treatment. Both forms of FGF-2 exhibited a protective effect with a non-significant difference between the LMW and HMW forms (Figure 1E). Moreover, we found both forms of FGF-2 increased Bcl-XL (an anti-apoptotic protein) transcript expression *via* the AKT signaling pathway in astrocytes (Figure 1F), suggesting the potential involvement of the anti-apoptotic protein Bcl-XL in the cytoprotection. We further analyzed oxidative stress status after various treatments in the astrocytes. As shown in Figures 1G,H, Aβ_1–42_ treatment significantly increased MDA level and decreased SOD activity in the conditioned medium of astrocytes, and LMW FGF-2 significantly decreased Aβ_1–42_-induced increased MDA concentration. However, LMW FGF-2 did not significantly increase Aβ_1–42_-induced decreased SOD activity in the astrocytes. In addition, the Aβ_1–42_-induced increased transcript expression of PGC-1, TFAM and NFκB were significantly decreased by LMW FGF-2 treatment in the astrocytes (Figures 1I–K).

### LMW FGF-2 Promotes Astrocyte Proliferation

**Figure 2:**
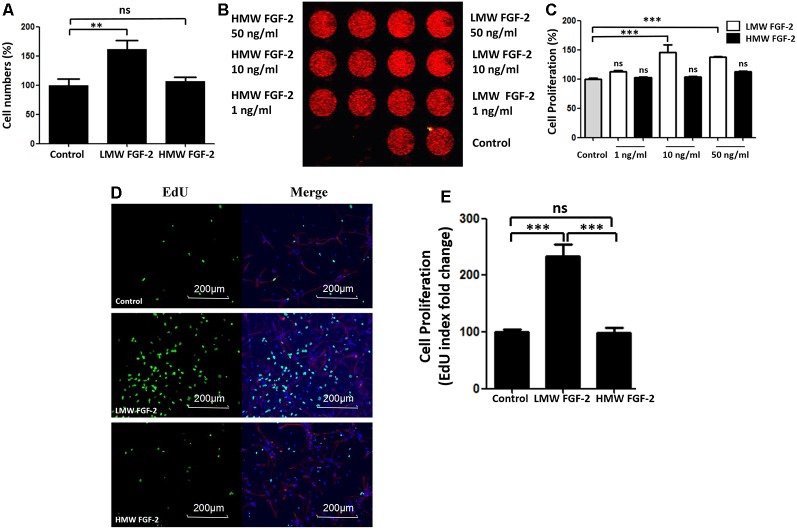
LMW FGF-2 promotes astrocyte proliferation. **(A)** LMW FGF-2 significantly increased cell numbers in the astrocytes as tested by trypan blue staining. **(B)** Fluorescence image of DRAQ5 showing DNA content, in astrocytes treated with or without FGF-2 at 1 ng/ml, 10 ng/ml and 50 ng/ml LMW or high molecular weight (HMW) FGF-2. **(C)** Quantification of DRAQ5 signal; 10 ng/ml and 50 ng/ml LMW FGF-2 treatments promote cell proliferation significantly, but not 1 ng/ml LMW FGF2. **(D)** Astrocytes with or without 10 ng/ml FGF-2 treatment stained by DAPI (blue), GFAP (red) and EdU (green). Note that the number of proliferating astrocytes (green) increases significantly after LMW FGF-2 treatment. **(E)** Quantification of EdU positive cells. Scale bar = 200 μm, *n* = 5. **p < 0.01, ***p < 0.001. ns, non-significance.

To determine whether FGF-2 promotes astrocyte proliferation, we incubated astrocytes with FGF-2 for 24 h and counted the cells after trypan blue staining. As shown in Figure 2A, 10 ng/ml LMW FGF-2 significantly promoted astrocyte proliferation compared to HMW FGF-2 and control group. We did DRAQ5 staining also to confirm proliferation. Fluorescent images show that there was more DNA stained with 10 and 50 ng/ml LMW FGF-2 treatment group than HMW FGF-2 treatment group and control group (Figure 2B). This red fluorescence signal was quantified, and the results show that 10 and 50 ng/ml LMW FGF-2 significantly promoted astrocyte proliferation compared with control (Figure 2C). Moreover, results from immunofluorescence also confirmed that LMW FGF-2, but not HMW FGF-2, promoted astrocyte proliferation, as evidenced by increased EdU staining, a nucleoside analog of thymidine and is incorporated into DNA during active DNA synthesis (Figures 2D,E).

### ERK and AKT Signaling Pathways Are Activated by FGF-2 in Astrocytes, but Only the AKT Signaling Pathway Was Required for FGF-2 Protective and Proliferative Functions

**Figure 3:**
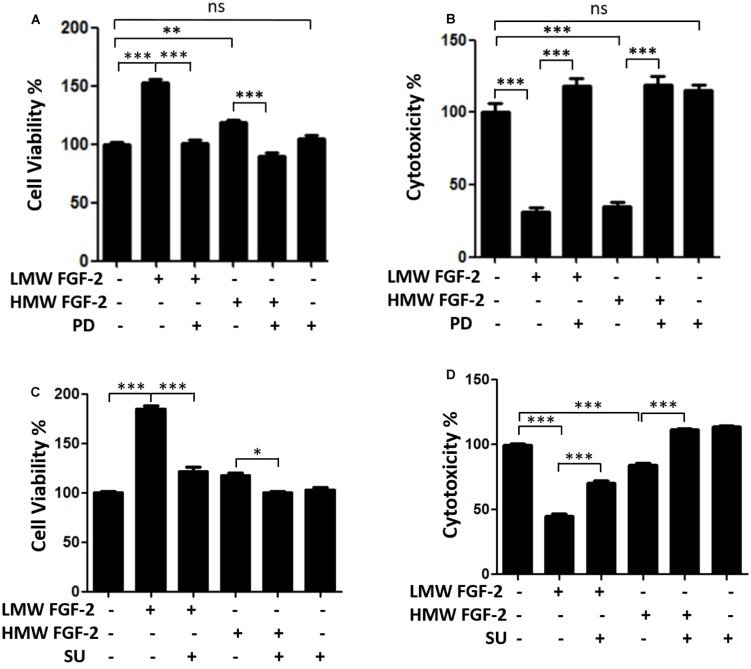
FGFR1 inhibitors block neuroprotective and proliferative effects of both isoforms FGF-2. **(A)** WST/MTT activity, indicating cell viability and proliferation of astrocytes treated with 10 ng/ml LMW and HMW FGF-2 and kinase inhibitor. Note that the cytoprotective and proliferative effects of both isoforms of FGF-2 were blocked by 0.1 μM PD166285. **(B)** LDH activity, indicating cytotoxicity of astrocytes treated with LMW and HMW FGF-2 and kinase inhibitor. Note that the cytoprotective effects of both isoforms FGF-2 were blocked by 0.1 μM PD166285. **(C)** WST/MTT activity, indicating cell viability and proliferation of astrocytes treated with LMW and HMW FGF-2 and kinase inhibitor. Note that neuroprotective and proliferative effects of both isoforms of FGF-2 were blocked by 10 μM SU5402. **(D)** LDH activity, indicating cytotoxicity of astrocytes treated with LMW and HMW FGF-2 and kinase inhibitor. Note that the neuroprotective effects of both isoforms of FGF-2 were blocked by 10 μM SU5402, *n* = 5. SU, SU5402. *p < 0.05, **p < 0.01, ***p < 0.001. ns, non-significance.

Figure 3 shows that the effects of LMW and HMW FGF-2 on astrocyte proliferation and survival were blocked by the FGFR1 inhibitors, PD166285, and SU5402. These results indicate that these functions of LMW or HMW FGF-2 were mediated by activating FGFR1.

**Figure 4:**
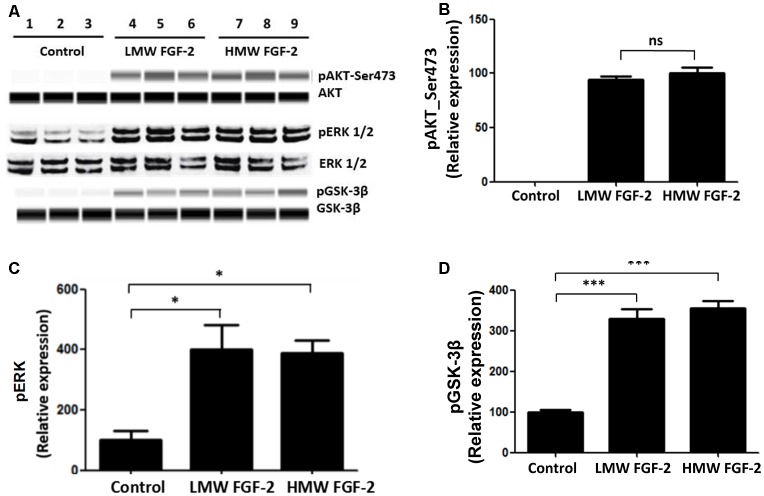
AKT, ERK, and GSK-3β are phosphorylated after FGF-2 treatment. **(A)** Western blot analysis showing that both LMW and HMW FGF-2 induce phosphorylation of AKT, ERK and GSK-3β in primary cultured astrocytes within 30 min treatment. **(B)** Western blot quantification of p-AKT (S473) signal in astrocytes. **(C)** Western blot quantification of p-ERK signal in astrocytes. **(D)** Western blot quantification of pGSK-3β signal in astrocytes. Data were normalized to the control group **(C,D)** or HMW FGF-2 treated group **(B)**, *n* = 3. *p < 0.05, ***p < 0.001. ns, non-significance.

FGF-2 is known to activate ERK and AKT signaling pathways (Cheng et al., 2016). Here, we showed that treatment of astrocytes with LMW and HMW FGF-2 for 30 min both resulted in increased phosphorylation of ERK, AKT (Ser473) and GSK-3β compared with the control group (Figure 4A). Thus, both LMW and HMW FGF-2 activated ERK and AKT (Ser473) signaling pathways similarly (Figures 4B–D).

**Figure 5:**
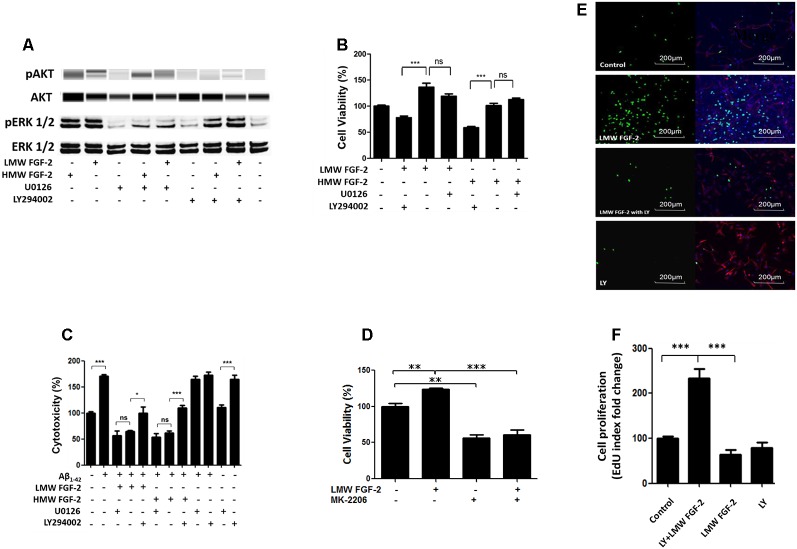
AKT signaling resulted in protective and proliferative effects of FGF-2. **(A)** Western blot analysis showing that FGF-2-induced phosphorylation of AKT and ERK in primary cultured astrocytes was blocked by LY294002 and U0126, respectively. U0126 is the ERK inhibitor; LY294002 is the AKT inhibitor. **(B)** WST/MTT activity in the primary cultured astrocytes treated with the two isoforms of FGF-2 and kinase inhibitors. Note that the survival or proliferative effects of FGF-2 were blocked by LY294002 in the primary cultured astrocytes, indicating that AKT signaling pathway is required for the protective effects of both LMW and HMW FGF-2. **(C)** LDH activity in the primary cultured astrocytes treated with Aβ_1–42_ (20 μM), two isoforms of FGF-2 and kinase inhibitors. Note that the protective effects of FGF-2 were blocked by LY294002 in the primary cultured astrocytes, indicating that AKT signaling pathway is required for the protective effects of LMW and HMW FGF-2 against Aβ_1–42_-induced cytotoxicity. **(D)** WST/MTT activity in the primary cultured astrocytes treated with LMW FGF-2 and MK-2206. **(E)** Astrocytes with or without LMW FGF-2 treatment and with continued presence or absence of LY294002 were stained by DAPI (blue), GFAP (red) and EdU (green). Note that the number of proliferating astrocytes (green) induced by FGF-2 decreases significantly after LY294002 treatment. **(F)** Quantification of EdU index fold change of different treatment, indicating astrocyte proliferation. Scale bar = 200 μm **(B–D)** *n* = 5; **(F)** *n* = 3. *p < 0.05, **p < 0.01, ***p < 0.001. ns, non-significance.

To determine whether the activation of ERK and AKT is required for the protective effects of FGF-2, we used the ERK inhibitor, U0126 and PI3K inhibitor, LY294002. Western blot data showed that the FGF-2-induced phosphorylation of ERK was blocked by 5 μM U0126 and that the FGF-2-induced phosphorylation of AKT was blocked by 10 μM LY294002, demonstrating the specificity of inhibitors (Figure 5A). Next, astrocytes were pretreated with U0126 and LY294002 for 30 min. Subsequently, 10 ng/ml HMW or LMW FGF-2 was added to the astrocytes for 24 h. As shown in Figure 5B, the increased cell viability in astrocytes by LMW FGF-2 was inhibited by LY294002, but not U0126. In addition, while FGF-2 still had a protective effect against Aβ_1–42_-induced cytotoxicity in the presence of U0126, LY294002 significantly inhibited the protective effect of FGF-2 against amyloid-beta toxicity (Figure 5C), indicating that only the AKT signaling pathway was required for the function of both isoforms of FGF-2. Interestingly, AKT inhibitor alone caused cytotoxicity in astrocytes, suggesting that endogenous AKT phosphorylation is important for cell survival. To further analyze the involvement of AKT signaling pathway in the observed effects of FGF-2 on astrocytes, we used another AKT inhibitor-MK-2206. The results showed that MK-2206 blocked the FGF-2-induced increased cell viability (Figure 5D), confirming that the AKT signaling pathway is important for the function of FGF-2 in astrocytes.

In addition, the function of AKT signaling in astrocyte proliferation was studied by treating astrocytes with the AKT inhibitor LY294002. The AKT inhibitor blocked LMW FGF-2-induced astrocyte proliferation as shown by a significantly decreased EdU staining (Figures 5E,F). Taken together, our results suggest that only the AKT signaling pathway is required for the protective effects of both LMW and HMW FGF-2, and the proliferative effect of LMW FGF-2.

### LMW FGF-2 Promotes Astrocyte Proliferation by Up-Regulation of c-Myc, Cyclin D1, and Cyclin E

**Figure 6:**
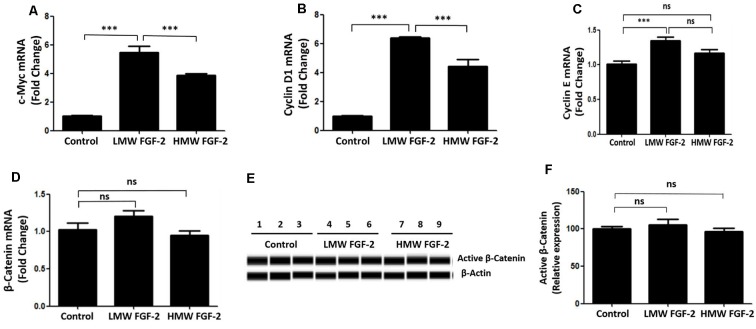
qRT-PCR analysis of c-Myc **(A)**, Cyclin D1 **(B)**, Cyclin E **(C)** and β-Catenin **(D)** expression in primary cultured astrocytes treated with or without LMW or HMW FGF-2 in serum-free media for 4 h. **(E)** Western blot showing active β-Catenin expression in primary cultured astrocytes treated with or without LMW or HMW FGF-2 in serum deprivation media for 24 h. **(F)** Western blot quantification of active β-Catenin signal, normalized to control group. Ccnd1, Cyclin D1, *n* = 3. ***p < 0.001. ns, non-significance.

**Figure 7:**
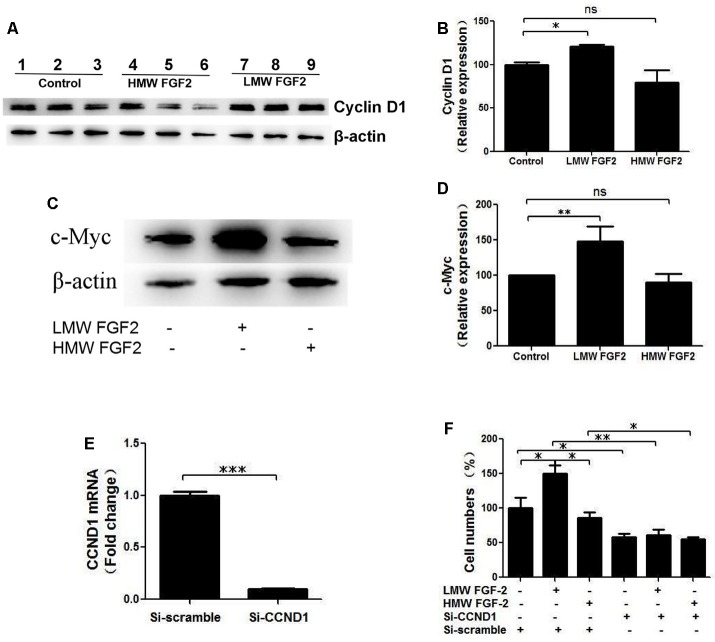
Cyclin D1 is crucial for FGF-2 induced astrocyte proliferation. **(A)** Western blot showing Cyclin D1 expression in primary cultured astrocytes treated with or without LMW or HMW FGF-2 in serum deprivation media for 24 h. **(B)** Western blot quantification of Cyclin D1 signal, normalized to control group. **(C)** Western blot showing c-Myc expression in primary cultured astrocytes treated with or without LMW or HMW FGF-2 in serum deprivation media for 24 h. **(D)** Western blot quantification of c-Myc signal, normalized to control group. **(E)** qRT-PCR analysis of Ccnd1 expression in primary cultured astrocytes treated with Ccnd1 siRNA or Si-scramble. **(F)** Ccnd1 siRNA treatment abolished LMW FGF-2-induced increased cell number in astrocytes as tested by trypan blue staining. Ccnd1, Cyclin D1. **(B)** *n* = 3; **(D)** *n* = 4; **(E–F)**, *n* = 5. *p < 0.05, **p < 0.01, ***p < 0.001. ns, non-significance.

qRT-PCR quantification of mRNA showed that both LMW and HMW FGF-2 significantly upregulated the expression of c-Myc and Cyclin D1, genes involved in cell cycle and proliferation. However, LMW FGF-2 was more potent to stimulate the two genes compared with HMW FGF-2 (Figures 6A,B). qRT-PCR quantification also indicated that only LMW FGF-2 significantly increased the expression of Cyclin E, a gene involved in cell cycle and proliferation (Figure 6C). the β-Catenin expression did not change after FGF-2 treatment in astrocytes, suggesting that the Wnt pathway is not involved in the FGF-2-mediated astrocyte proliferation (Figure 6D). In addition, active β-Catenin protein levels were not changed after FGF-2 treatment in astrocytes (Figures 6E,F). We further showed that LMW FGF-2, but not HMW FGF-2, increased Cyclin D1 and c-Myc protein levels in the astrocytes (Figures 7A–D). To investigate whether Cyclin D1 mediated the FGF-2-induced astrocyte proliferation, we silenced the Cyclin D1 gene expression in astrocytes (Figure 7E) and counted the cell numbers after various treatments. Treatment with Cyclin D1 siRNA in astrocytes abolished FGF-2-induced increased cell number (Figure 7F), demonstrating that Cyclin D1 is required for the proliferative effect of FGF-2 in astrocytes.

## Discussion

Previous studies have demonstrated that FGF-2 has a neuroprotective effect by promoting neuronal progenitor cell differentiation and neurogenesis in the dentate gyrus (Naruse et al., 2015). Diminished neuronal FGF-2 levels are linked to neurological diseases including AD (Woodbury and Ikezu, 2014) and depression (Cheng et al., 2015). Moreover, FGF-2 has been demonstrated to be a potential therapeutic agent for treating these diseases (Katsouri et al., 2015). While the expression of FGF-2 in neurons is more restricted to certain brain areas such as the CA2 region of the hippocampus (Weickert et al., 2005), astrocytes are known to be a major source of FGF-2 in central nervous system (Goddard et al., 2002), yet the role of different forms of FGF-2 on astrocytes remains poorly understood. Early studies have shown that FGF-2 is secreted from astrocytes and has an autocrine function in promoting its own expression and proliferation (Gómez-Pinilla et al., 1995; Delgado-Rivera et al., 2009). In this study, we have investigated the protective and proliferative effect of LMW (17 kDa) and HMW (23 kDa) FGF-2 on astrocytes. LMW FGF-2 is secreted from astrocytes (Delgado-Rivera et al., 2009) but it is unclear if the HMW form is also secreted. We found that both isoforms had similar protective effects against Aβ_1–42_-induced cytotoxicity and oxidative stress in astrocytes. However, only LMW FGF-2 promoted astrocyte proliferation. We also found that FGF-2 activated ERK and AKT signaling pathways. However, only the AKT signaling pathway was required for the neuroprotective and proliferative effects of FGF-2. Further results suggested that LMW FGF-2 was more potent, compared to HMW FGF-2, in upregulating the expression of c-Myc, Cyclin D1 and Cyclin E.

Differences in function between LMW and HMW FGF-2 may be due to the three-dimensional structure of the two forms, which in turn may have differential effects on cell surface receptor stimulation. There is some evidence that extracellular-acting HMW FGF-2, *via* its N-terminal extension, could engage a co-receptor, neuropilin 1, in addition to the FGFR, at the cell surface (Zhang et al., 2013). Therefore, it is possible that a co-receptor only activated by one type of FGF-2 isoform may explain the differential effects of LMW vs. HMW FGF-2 on cell proliferation.

**Figure 8:**
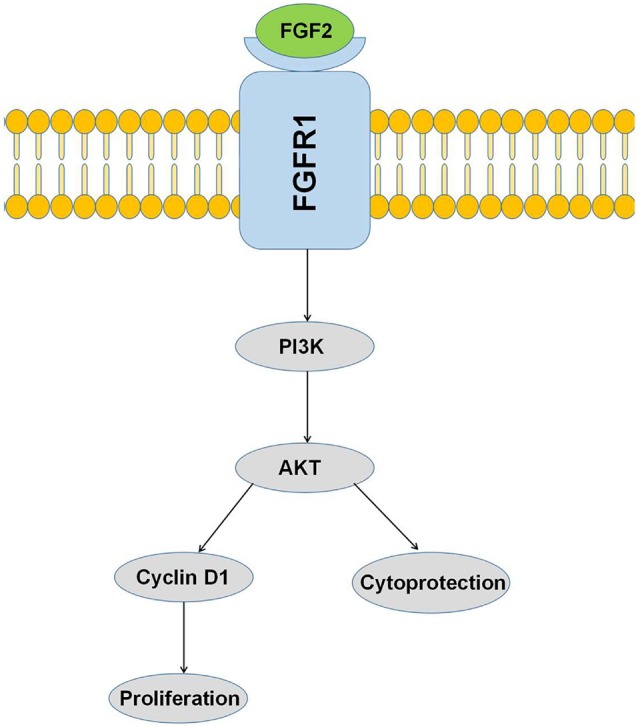
Activation of the PI3K/AKT pathway by FGF-2 leads to cytoprotection and proliferation of astrocytes.

Cyclin D1 functions as a regulatory subunit of CDK4 or CDK6, and the activity is required for the cell cycle progression through G1 phase (Chen et al., 2018). Cyclin E forms a complex with CDK2 and plays a critical role in the transition from G1 to S phase, thus regulating proliferation (Sun et al., 2015). c-Myc regulates gene expression through the binding on the Enhancer Box sequence (E-boxes) and recruiting histone acetyltransferases (Xiong et al., 2010). Our results demonstrated that LMW FGF-2 up-regulated Cyclin D1, Cyclin E and c-Myc expression significantly, although HMW FGF-2 treatment also increased Cyclin D1 and c-Myc mRNA expression but to a lesser extent. This may partly explain the differential proliferative effects between LMW and HMW FGF-2. Indeed, knocking down Cyclin D1 gene expression abolished LMW FGF-2-mediated astrocyte proliferation. This suggests the important role of Cyclin D1 in LMW FGF-2-induced astrocyte proliferation. In summary, we have found that both 17 KDa and 23 KDa isoforms of FGF-2 effectively protected astrocytes against Aβ _1–42_- and oxidative stress-induced cytotoxicity *via* the AKT signaling pathway (Figure 8). Furthermore, while LMW FGF-2 promoted astrocyte proliferation *in vitro*, HMW FGF-2 did not. These findings showing differential effects of the two forms of FGF-2 on astrocyte proliferation could be of major importance in facilitating the tailoring of the forms of FGF-2 to be used as a potential therapeutic agent for treatment of AD. Indeed, FGF-2 induced astrogliosis has been suggested to be associated with central nervous system damage (Goddard et al., 2002). In addition, it is known that FGF-2 secreted from astrocytes can stimulate cancer cell proliferation (Placone et al., 2016). Hence having the 23 KDa form of FGF-2 that has neuroprotective but no proliferative action could be advantageous as a potential therapeutic agent. Future work extending these studies to further investigate the effect of using HMW FGF-2 *in vivo* will determine if it can be used to treat neurodegenerative diseases such as AD.

## Data Availability Statement

The raw data supporting the conclusions of this article will be made available by the authors, without undue reservation, to any qualified researcher.

## Ethics Statement

This study was carried out in accordance with the guidelines approved by the Animal Care and Use Committee NICHD, NIH, and the Animal Care and Use Committee of Minzu University of China.

## Author Contributions

YC and YL conceived and designed the study. XC and ZL performed the experiments. ZL drafted the manuscript with critical revisions from XC, YC, EK, and YL. EK provided biologically active recombinant HMW-FGF2 and LMW-FGF2 proteins, and critical feedback on the draft manuscript.

## Conflict of Interest

The authors declare that the research was conducted in the absence of any commercial or financial relationships that could be construed as a potential conflict of interest.

## Footnotes

^1^http://www.proteinsimple.com/wes.html
